# Mortality associated with neurofibromatosis type 1: A study based on Italian death certificates (1995-2006)

**DOI:** 10.1186/1750-1172-6-11

**Published:** 2011-03-25

**Authors:** Maria Masocco, Yllka Kodra, Monica Vichi, Susanna Conti, Mark Kanieff, Monica Pace, Luisa Frova, Domenica Taruscio

**Affiliations:** 1 National Centre for Epidemiology, Surveillance and Health Promotion, Italian National Institute of Health (ISS), Rome, Italy; 2 National Centre for Rare Diseases, Italian National Institute of Health (ISS), Rome, Italy; 3 Division for Statistics and Surveys on Social Institutions, Italian National Institute of Statistics (ISTAT), Rome, Italy

## Abstract

**Background:**

Persons affected by neurofibromatosis type 1 (NF1) have a decreased survival, yet information on NF1-associated mortality is limited.

**Methods/Aim:**

The National Mortality Database and individual Multiple-Causes-of-Death records were used to estimate NF1-associated mortality in Italy in the period 1995-2006, to compare the distribution of age at death (as a proxy of survival) to that of the general population and to evaluate the relation between NF1 and other medical conditions by determining whether the distribution of underlying causes of NF1-associated deaths differs from that of general population.

**Results:**

Of the nearly 6.75 million deaths in the study period, 632 had a diagnosis of NF1, yet for nearly three-fourths of them the underlying cause was not coded as neurofibromatosis. The age distribution showed that NF1-associated deaths also occurred among the elderly, though mortality in early ages was high. The mean age for NF1-associated death was approximately 20 years lower than that for the general population. The gender differential may suggest that women are affected by more severe NF1-related complications, or they may simply reflect a greater tendency for NF1 to be reported on the death certificates of young women. Regarding the relation with other medical conditions, we found an excess, as the underlying cause of death, for malignant neoplasm of connective and other soft tissue and brain, but not for other sites. We also found an excess for obstructive chronic bronchitis and musculoskeletal system diseases among elderly persons.

**Conclusion:**

This is the first nationally representative population-based study on NF1-associated mortality in Italy. It stresses the importance of the Multiple-Causes-of-Death Database in providing a more complete picture of mortality for conditions that are frequently not recorded as the underlying cause of death, or to study complex chronic diseases or diseases that have no specific International Classification of Diseases code, such as NF1. It also highlights the usefulness of already available data when a surveillance system is not fully operational.

## Background

Neurofibromatosis type 1 (NF1) is an autosomal dominant disorder which affects 1 in 2500-3000 live births [1,2]. It is a progressive multisystem disorder characterized by a wide variety of clinical signs and symptoms, a totally unpredictable evolution, and an increased risk of malignancy. The gene responsible for NF1 encodes neurofibromin, a protein which acts as a tumor suppressor, the loss of which leads to an increased risk of developing tumors. The hallmark feature of NF1 is benign neurofibromas (a peripheral nerve sheath tumor); the other NF1-defining manifestations consist of cafè au lait patches, skin-fold freckling, iris Lisch nodules, optic pathway glioma, and bony dysplasia [3]. NF1 can also result in serious complications which affect diverse body systems and which are responsible for the deaths related to NF1; these include disfigurement, evolving scoliosis, cognitive or neurological impairment, vasculopathy, and malignancy, in particular, malignant tumors of peripheral and central nerve tissue [4-11].

Although persons with NF1 have a decreased survival, information on NF1-associated mortality is limited. Only one nationally representative population-based study on NF1 mortality based on death certificates has been published [12], and the information on the causes of death among persons with NF1 mainly derives from case series. Moreover, given that NF1 has a variety of clinical manifestations and complications of varying degrees of severity, it may not be recorded as the official cause of death. An additional problem is that no specific code exists for NF1 in the International Classification of Diseases (ICD), so that it is not possible to distinguish it from other types of neurofibromatosis, such as neurofibromatosis type 2 (NF2). The importance of this distinction lies in the fact that NF1 and NF2 are clinically and genetically different, generally with different symptoms and requiring distinct medical management; nonetheless, they are often confused for one another by clinicians. Although NF2 is more aggressive, NF1 is numerically more important, in that its incidence is about 10 times greater than that of NF2 [13].

The objective of this study was to estimate NF1-associated mortality in Italy, in particular, the number of NF1-associated deaths in the past 12 years; we also compared the distribution of age at death to that of the general population, so as to have an indication of survival, and explored the relation between NF1 and other medical conditions.

## Methods

### Data sources

Two data sources were used: the National Mortality Database, and individual Multiple-Causes-of-Death records, provided by Italy's National Institute of Statistics (ISTAT). The National Mortality Database contains the underlying cause of death for all persons who died from 1970 to 2006 (most recent data available), which is coded by ISTAT. Until 2002, the 9^th ^Revision of the ICD (ICD-9) was used; since then, ICD-10 has been used; however, in 2004 and 2005, the underlying cause was not codified. A single code is used for all types of neurofibromatoses: 237.7 in ICD-9 and Q85.0 in ICD-10.

The Multiple-Causes-of-Death records contain all causes and other important conditions as reported by the certifying physician on the death certificate, they are available for the period 1995-2006 and cover the entire country, with the exception of residents in the provinces of Trento and Bolzano and infants (less than one year of age); for infants, Multiple-Causes-of-Death records have been available since 2003. All of the conditions and diseases reported on the medical part of the death certificate are entered in the database by data-entry specialists; this step is supported by a dictionary of medical terms which provides assistance in case of incorrect entries.

All deaths in 1995-2003 and 2006 that were coded as 237.7 or Q85.0 were selected from the National Mortality Database; to obtain information on all of the diagnoses for these individuals, these deaths were linked to the Multiple-Causes-of-Death records, using the anonymous individual identification code used by both systems. Moreover, all Multiple-Causes-of-Death records with neurofibromatosis reported anywhere on the death certificate (as underlying or other cause) were selected by searching for the term "neurofibromatosis" or any synonym, acronym or short form of "neurofibromatosis" or "Von Recklinghausen" disease; the search was expanded to include symptoms of NF1 (complete term, synonym, or acronym), according to published diagnostic criteria [3].

We then excluded all Multiple-Causes-of-Death records with the following:

• the term "neurofibroma" without any other indication of neurofibromatosis (16 cases)

• an evident diagnosis of Neurofibromatosis type 2 (NF2) (17 cases)

• a diagnosis of neurofibromatosis (type not specified) with acoustic neurinoma or meningyoma (or synonyms), which are more likely to be associated with NF2 (6 cases).

We also included the three deaths in the provinces of Trento and Bolzano (all three from Bolzano) for which the underlying cause was coded as 237.7 or Q85.0 (not included in the Multiple-Causes-of-Death database). With the exception of these three deaths, all deaths coded as 237.7 or Q85.0 in the National Mortality Database were present in the Multiple-Causes-of-Death records. Similarly, all deaths selected from the Multiple-Causes-of-Death database with a diagnosis of NF1 were present in the National Mortality Database, although for the 101 deaths in the period 2004-2005, only demographic data were available in the National Mortality Database whereas the underlying cause was not coded.

Table 1 shows the diagnosis written out on death certificates of the individuals included in the analysis.

**Table 1:** Diagnosis reported on the death certificates of persons identified as having neurofibromatosis type 1

Alphanumeric string on death certificate	No. of deaths
*"Von Recklingausen" *disease	344

*"Neurofibromatosis" *not otherwise specified (without mention of acoustic Neurinoma and/or Meningyoma)	215

*"Diffuse Neurofibromatosis"*	33

*"Neurofibromatosis Type 1"*	24

*"Cutaneous Neurofibromatosis"*	7

*"Visceral or Retroperitoneal Neurofibromatosis"*	3

*"Cerebral Neurofibromatosis" *(not otherwise specified and without any symptoms)	3

Deaths from National Mortality Database but not present in Multiple-Causes-of-Death records (deaths from Trento and Bolzano)	3

**Total**	**632**

### Statistical analyses

We calculated the mean annual NF1-associated mortality by age, using the Italian population at 1 January 2001 [14] as a proxy of the average annual person-years in 1995-2006. We also estimated the mean annual NF1-associated mortality for men and women, adjusted by age and sex, using direct method and the Italian population at 1 January 2001 as standard population. To evaluate gender differences, we calculated the standardized Rate Ratio (RR), as the ratio of male/female standardized rates, with relative 95% confidence intervals (CI) [15].

To approximate the survival of persons with NF1, we compared the age distribution of NF1-associated deaths with the age distribution of persons who died from all causes in the same period.

For NF1-associated deaths and persons who died from all causes, the mean and median age at death, and the 5th, 25th, 75th, 95th percentiles of age distribution were calculated overall, by gender, and, to investigate temporal differences, for the periods 1995-2000 and 2001-2006. Differences in mean age at death, and relative 95% CI, between NF1-associated deaths and deaths from all causes were calculated.

To investigate whether or not the effect of NF1-associated mortality was restricted to younger patients, the analyses were repeated for persons who were 40 years old or older.

Non-parametric methods (U Mann-Whitney test, Median test) were used to test the gender and temporal differences in mean ages at death of NF1-associated deaths, because the age distribution of these cases was skewed and bi-modal (p < 0.001: Kolmogorov-Smirnov test for normality distribution). Student-t test was used to evaluate gender and temporal differences in mean age at death among the general population (deaths from all causes). The analyses were performed using SPSS software (version 17).

To investigate the potential association between NF1 and other conditions, we determined whether the distribution of the underlying causes of NF1-associated deaths differed from that of the general population by calculating the Proportionate Mortality Ratio (PMR). For each condition (or group of conditions) reported as underlying cause of death for NF1-associated deaths, the PMR was calculated as the ratio of observed deaths to expected deaths; the expected deaths were based on the proportion of deaths due to the given condition among the same-age general population of persons who died in the same calendar period. An excess of a given condition among deceased persons with NF1 is indicated by a PMR >1, whereas a deficit is indicated by a PMR <1. The 95% CI of PMRs were calculated using the Byar's formula as an approximation of the exact Poisson test [16].

## Results

### NF1-associated deaths

Of the nearly 6 753 000 deaths in 1995-2006, 632 had a diagnosis of NF1 on the death certificate (herein defined as "NF1-associated deaths"); of these, 150 had neurofibromatosis as the underlying cause of death (ICD-9: 237.7; ICD-10: Q85.0).

The mean annual frequency of NF1-associated deaths was 1/10 685 deaths, and the mean annual NF1-associated mortality was 0.92 per 1 million population. The age-sex-adjusted mortality was 1.01 for men (95% CI: 0.62-1.40) and 0.85 for women (95% CI: 0.52-1.17) (RR = 1.19, 95% CI: 0.69-2.06).

**Figure 1:**
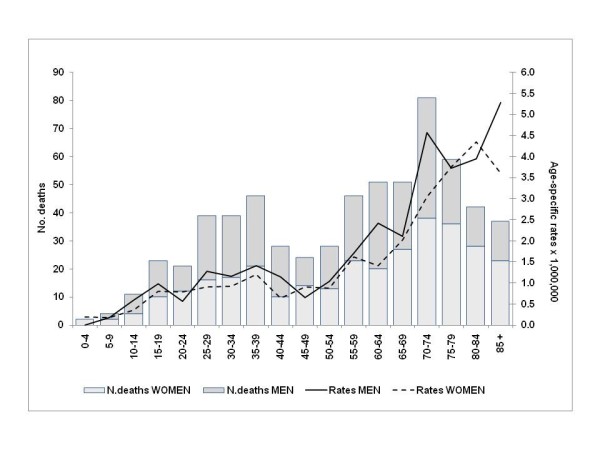
**Annual average NF1-associated mortality (per 1,000,000 population) and total number of deaths, by age; 1995-2006**.

Mortality increased with age, yet premature mortality was high, and 185 of the 632 deaths identified (30%) were at ages younger than 40 years (Figure 1). In particular, age-specific mortality rates increased, reaching a relative maximum at 40 years; in the successive 10 years it then decreased, reaching the same levels found among adolescents; finally, after the age of 50 years, it began to increase continuously.

**Figure 2:**
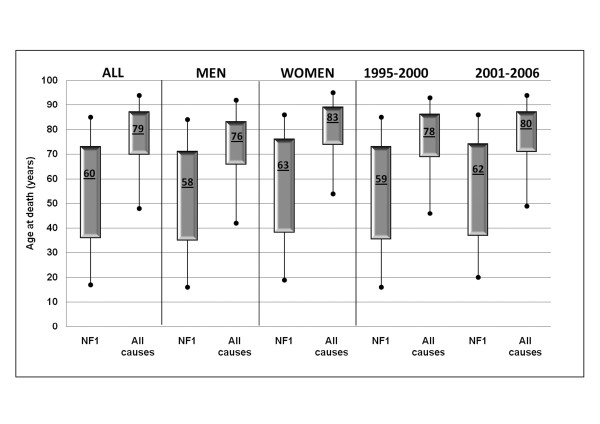
**Median (in box) and 5^th^, 25^th^, 75^th ^and 95^th ^percentiles of age at death**.

The distribution of NF1-associated deaths was more spread out than that for deaths for all causes (Figure 2). NF1-associated deaths were, on average, younger than deaths for all causes, for both men and women. Of the NF1-associated deaths, 5% occurred among persons younger than 17 years of age and 25% among persons younger than 36 years, whereas among the general population, 5% of deaths occurred among persons younger than 50 years and 25% among persons younger than 70 years. The median age of NF1-associated death was 60 years, compared to 79 years for the general population; the difference in median age of death was slightly larger among women, compared to men.

The mean age at death for NF1-associated deaths was 55.5 years, compared to 76.2 years for the general population; it was greater among women than among men, yet the gender difference, although significant, was smaller compared to that of the general population (Table 2). From 1995-2002 to 2001-2006, there was an increase in the mean age of NF1-associated deaths, and this increase was greater than that observed for the general population.

**Table 2:** Mean age at death and Standard Deviation (SD) for NF1-associated deaths and all causes (among the general population), by gender, calendar period and age class; Italy, 1995-2006

**ALL AGES**	**NF1-associated deaths**	**Mean age at death (SD)**				**Differences in mean age at death (95% CI)**	
			
		**NF1**		**All causes**		**NF1 vs. All causes**	
			
**Total**	632	55.5	*(21.8)*	76.2	*(15.2)*	20.7	(19.0 - 22.4)
			
**Men**	316	53.3	*(21.2)*	72.8	*(15.6)*	19.5	(17.2 - 21.9)
			
**Women**	316	57.7	*(22.1)*	79.5	*(14.0)*	21.8	(19.4 - 24.3)
			
***Gender difference***		*4.4*	*p ≤ 0.004*	*6.7*	*p ≤ 0.0001*		
							
			
**1995-2000**	329	54.4	*(22.0)*	75.3	*(15.5)*	20.9	(18.5 - 23.3)
			
**2001-2006**	303	56.7	*(21.5)*	77.0	*(14.8)*	20.3	(17.9 - 22.7)
			
***Temporal difference***		*2.4*	*n.s.*	*1.8*	*p ≤ 0.0001*		
							
**AGES ≥ 40 years**	**NF1-associated deaths**	**Mean age at death (SD)**				**Differences in mean age at death (95% CI)**	
		**NF1**		**All causes**		**NF1 vs. All causes**	
			
**Total**	447	67.3	(12.9)	77.9	(11.9)	10.6	(9.4 - 11.8)
			
**Men**	215	65.5	(12.6)	75.0	(11.8)	9.5	(7.8 - 11.2)
			
**Women**	232	68.9	(13.0)	80.7	(11.3)	11.8	(10.1 - 13.5)
			
***Gender difference***		*3.4*	*p ≤ 0.004*	*5.7*	*p ≤ 0.0001*		
							
			
**1995-2000**	230	66.6	(12.9)	77.2	(11.8)	10.7	(9.0 - 12.3)
			
**2001-2006**	217	68.0	(12.9)	78.5	(11.9)	10.5	(8.8 - 12.2)
			
***Temporal difference***		*1.4*	*n.s.*	*1.3*	*p ≤ 0.0001*		

When restricting the analysis to persons 40 years of age and older, the gap in the mean age of death between NF1-cases and the general population was 10.6 years (mean age of death of 67.3 years vs. 77.9 years) (Table 2).

### Excesses and deficits of underlying causes

The estimated PMR for the major groups of underlying causes for NF1-associated deaths are shown in Table 3; the results refer to the 531 deaths that occurred in 1995-2003 and 2006. For approximately 72% of the NF1-associated deaths, the underlying cause was coded as a pathology other than neurofibromatosis. Of these causes, neoplasms (ICD-9 140-239, excluding 237.7; ICD-10 C00-D48) were the most frequent, accounting for one third of all NF1-associated deaths.

**Table 3:** NF1-associated deaths by underlying cause

	Number		PMRs	95% CI
			
Underlying cause of death (ICD-9 and ICD-10 Codes )	**Observ**.	**Expect**.		
			
Neurofibromatosis (ICD-9: 237.7; ICD-10: Q85.0)	150	-		
Neoplasms, excluding neurofibromatosis (ICD-9: 140-239, excluding 237.7; ICD-10: C00-D48)	182	175.5	1.0	(0.89 - 1.20)

Malignant neoplasms (ICD-9:140-208; ICD10: C00-C97)	158	169.0	0.9	(0.79 - 1.09)
Lip, oral cavity and pharynx (ICD-9:140-149; ICD10: C00-C14)	1	4.1	0.2	(0.00 - 1.37)
Colorectal (ICD-9:153-154; ICD-10: C18-C21)	9	15.6	0.6	(0.26 - 1.09)
Stomach (ICD-9:151; ICD10: C16)	2	10.9	0.2	(0.02 - 0.66)
Liver and intrahepatic bile ducts (ICD-9:155; ICD10: C22)	1	9.9	0.1	(0.00 - 0.56)
Pancreas (ICD-9:157; ICD10: C25)	5	7.9	0.6	(0.20 - 1.47)
Trachea/bronchus/lung (ICD-9:162; ICD-10: C33-C34)	11	34.3	0.3	(0.16 - 0.57)
Bone and articular cartilage (ICD-9:170; ICD-10: C40-C41)	3	1.3	2.3	(0.45 - 6.59)
Connective and other soft tissue (ICD-9:171; ICD-10: C49)	35	1.6	22.3	(15.50 - 30.95)
Skin (malignant melanoma) (ICD-9:172: ICD-10: C43)	4	2.6	1.5	(0.41 - 3.89)
Breast (ICD-9:174-175; ICD-10: C50)	6	15.2	0.4	(0.14 - 0.86)
Body (or unspecified part) of uterus (ICD-9:179; 182; ICD-10: C54-C55)	1	2.6	0.4	(0.01 - 2.15)
Cervix uteri (ICD-9:180; ICD-10: C53)	-	-	-	-
Ovary (ICD-9:183.0; ICD-10: C56)	2	3.5	0.6	(0.06 - 2.04)
Prostate (ICD-9:185; ICD-10: C61)	1	4.2	0.2	(0.00 - 1.31)
Kidney, except pelvis (ICD-9:189.0; ICD-10: C64)	2	3.2	0.6	(0.07 - 2.27)
Bladder (ICD-9:188; ICD-10: C67)	4	3.9	1.0	(0.28 - 2.65)
Brain (ICD-9:191; ICD-10: C71)	25	6.0	4.2	(2.69 - 6.15)
Other and unspecified parts of nervous system (ICD-9:192; ICD-10: C70; C72)	4	0.2	20.4	(5.50 - 52.35)
Other and ill-defined sites (ICD-9:195; ICD-10: C76)	4	1.0	4.2	(1.13 - 10.72)
Neoplasm without specification of site (ICD-9:199; ICD-10: C80)	9	4.6	2.0	(0.89 - 3.72)
Lymphatic and hematopoietic tissue (ICD-9: 200-208; ICD-10: C81-C96)	5	18.3	0.3	(0.09 - 0.64)
Malignant neoplasms (excluding those of connective and soft tissue, brain, other and unspecified parts of nervous system, or other and ill-defined sites)	90	160.2	0.6	(0.45 - 0.69)
Benign Neoplasms (ICD-9:210-229; ICD-10: D10-D36)	7	0.8	9.2	(3.69 - 18.99)
Neoplasms of uncertain behavior and unspecified nature, excluding neurofibromatosis (ICD-9: 235-239, excluding 237.7; ICD-10: D37-D48)	17	5.8	2.9	(1.72 - 4.72)

Infectious and parasitic diseases, including AIDS (ICD-9: 1-139, 279.1; ICD-10: A00-B99)	5	17.5	0.3	(0.09 - 0.67)

Endocrine, nutritional and metabolic diseases (ICD-9: 240-278 ICD-10: E00-E90)	9	15.9	0.6	(0.26 - 1.07)
Diabetes mellitus (ICD-9: 250; ICD-10: E10-E14)	2	12.3	0.2	(0.02 - 0.59)

Diseases of the blood and blood-forming organs and certain disorders involving the immune mechanism, excluding AIDS (ICD-9: 279-289; excluding 279.1; ICD-10: D50-D89)	2	2.6	0.8	(0.09 - 2.78)

Mental disorders (ICD-9: 290-319; ICD-10: F00-F99)	1	10.1	0.1	(0.00 - 0.55)

Diseases of the nervous system and sense organs (ICD-9: 320-389; ICD-10: G00-H95)	10	13.8	0.7	(0.35 - 1.33)

Diseases of the circulatory system (ICD-9: 390-459; ICD-10: I00-I99 )	101	143.5	0.7	(0.57 - 0.86)
Hypertensive disease (ICD-9: 401-405; ICD-10: I10-I15)	6	9.9	0.6	(0.22 - 1.32)
Ischemic heart disease (ICD-9: 410-414; ICD-10: I20-I25)	23	51.0	0.5	(0.29 - 0.68)
Cerebrovascular disease (ICD-9: 430-438; ICD-10: I60-I69)	31	35.4	0.9	(0.59 - 1.24)

Diseases of the respiratory system (ICD-9: 460-519; ICD-10: J00-J99)	33	21.4	1.5	(1.06 - 2.17)
Obstructive chronic bronchitis (ICD-9: 491.2; ICD-10: J448)	13	6.5	2.0	(1.06 - 3.40)

Diseases of the digestive system (ICD-9: 520-579; ICD-10: K00-K93 )	14	24.8	0.6	(0.31 - 0.95)

Diseases of the genitourinary system (ICD-9: 580-629; ICD-10: N00-N99)	3	4.9	0.6	(0.12 - 1.78)

Diseases of the skin and subcutaneous tissue (ICD-9: 680-709; ICD-10: L00-L99)	2	0.4	5.3	(0.59 - 18.98)

Diseases of the musculoskeletal system and connective tissue (ICD-9:710-739; ICD-10: M00-M99)	5	1.8	2.7	(0.88 - 6.34)

Congenital anomalies, excluding neurofibromatosis (ICD-9:740-759; ICD-10: Q00-Q99, excluding Q85.0)	3	3.6	0.8	(0.17 - 2.44)

Injury and poisoning (ICD-9: 800-999; ICD-10: S00-T98)	11	83.6	0.1	(0.07 - 0.24)

**TOTAL**	531			

Italy, 1995-2003 and 2006.

According to the PMR, with regard to malignant tumors, a 22-fold excess was found for connective and other soft tissue (PMR = 22.3, 95% CI: 15.50-30.95), and a 4-fold excess for brain (PMR = 4.2, 95% CI: 2.69-6.15). A 20-fold excess was also found for other and unspecified parts of the nervous system (other than brain) (PMR = 20.4, 95% CI: 5.50-52.35) and a 4-fold excess for other and ill-defined sites (PMR = 4.2, 95% CI: 1.13-10.72), though they referred to fewer cases. When excluding the above malignant neoplasms (neoplasm of connective and soft tissue, brain, unspecified parts of the nervous system and ill-defined sites), there was a significant deficit for malignant neoplasms as a group (PMR = 0.6, 95% CI: 0.45-0.69). There were also significant excesses for benign neoplasms (PMR = 9.2, 95% CI: 3.69-18.99) and neoplasms of uncertain behavior and unspecified nature (other than neurofibromatosis) (PMR = 2.9, 95% CI: 1.72-4.72).

Of the non-neoplastic diseases, a slight excess was found for those of the respiratory system as a whole, which can be attributed to the excess found for obstructive chronic bronchitis (PMR = 2.0, 95% CI: 1.06-3.40).

The estimated PMRs stratified by age are shown in Table 4. For persons who died before the age of 40 the PMRs were higher with respect to those for all ages for malignant neoplasm of connective and soft tissue, brain, unspecified parts of the nervous system and without specification of the sites and for benign neoplasms and neoplasms of uncertain behavior, which resulted in an increase in PMRs for all neoplasms as a group and malignant neoplasms as a group (PMR = 2.9, 95% CI: 2.33-3.68 and PMR = 2.8, 95% CI: 2.15-3.51, respectively). By contrast, the PMR for respiratory diseases was no longer significant. Among persons who died at 40 years of age or older, the PMRs continued to be significant only for malignant neoplasm of connective and soft tissue, benign neoplasms and neoplasms of uncertain behavior and for diseases of the respiratory system; a significant excess was also found for diseases of the musculoskeletal system (PMR = 3.6, 95% CI: 1.15-8.34).

**Table 4:** NF1-associated deaths by selected underlying cause, stratified by age

People died at age < 40 years				
	Number			
			
Underlying cause of death (ICD-9 and ICD-10 Codes )	**Observ**.	**Expect**.	PMRs	95% CI
	
Neurofibromatosis (ICD-9: 237.7; ICD-10: Q85.0)	59	-		
Neoplasms, excluding neurofibromatosis (ICD-9: 140-239, excluding 237.7; ICD-10: C00-D48)	77	26.1	2.9	(2.33 - 3.68)
Malignant Neoplasms (ICD-9: 140-208; ICD-10: C00-C97)	68	24.6	2.8	(2.15 - 3.51)
Connective and other soft tissue (ICD-9:171; ICD-10: C49)	21	0.9	23.4	(14.45 - 35.71)
Brain (ICD-9:191; ICD-10: C71)	19	2.5	7.5	(4.50 - 11.67)
Other and unspecified parts of nervous system (ICD-9:192; ICD-10: C70; C72)	3	0.1	32.7	(6.58 - 95.68)
Neoplasm without specification of site (ICD-9:199; ICD-10: C80)	4	0.6	6.8	(1.82 - 17.37)
Benign Neoplasms (ICD-9:210-229; ICD-10: D10-D36)	2	0.2	11.0	(1.23 - 39.54)
Neoplasms of uncertain behavior and unspecified nature, excluding neurofibromatosis (ICD-9: 235-239, excluding 237.7; ICD-10: D37-D48)	7	1.4	5.1	(2.04 - 10.50)

**TOTAL**	151			
**People died at age ≥ 40 years**				
	**Number**			
			
**Underlying cause of death (ICD-9 and ICD-10 Codes )**	**Observ**.	**Expect**.	**PMRs**	**95% CI**
	
Neurofibromatosis (ICD-9: 237.7; ICD-10: Q85.0)	91	-		

Connective and other soft tissue (ICD-9:171; ICD-10: C49)	14	0.7	20.8	(11.35 - 34.87)
Benign Neoplasms (ICD-9:210-229; ICD-10: D10-D36)	5	0.6	8.7	(2.79 - 20.22)
Neoplasms of uncertain behavior and unspecified nature, excluding neurofibromatosis (ICD-9: 235-239, excluding 237.7; ICD-10: D37-D48)	10	4.4	2.3	(1.09 - 4.19)

Diseases of the respiratory system (ICD-9: 460-519; ICD-10: J00-J99)	30	18.2	1.6	(1.11 - 2.35)
Obstructive chronic bronchitis (ICD-9: 491.2; ICD-10: J44.8)	13	6.4	2.0	(1.08 - 3.47)

Diseases of the musculoskeletal system and connective tissue (ICD-9:710-739; ICD-10: M00-M99)	5	1.4	3.6	(1.15 - 8.34)

**TOTAL**	380			

Italy, 1995-2003 and 2006.

## Discussion

Of the nearly 6 753 000 deaths in Italy in 1995-2006, 632 had a diagnosis of NF1 on the death certificate, yet for nearly three-fourths of them the underlying cause was coded as another pathology.

With regard to age, the bimodal trend in NF1-associated mortality emphasizes the weight of premature mortality (i.e., before 40 years of age): mortality dramatically increased from adolescence to 40 years of age, with one third (n = 185) of the deaths occurring before 40 years; after 40 years, the mortality decreased until 50 years of age and then showed a constant increase for older ages, with the highest absolute number of deaths among 70-74 year olds.

Compared to the general population, the mean age of NF1-associated deaths was approximately 20 years lower, and it was approximately 10 years lower when the analysis was restricted to persons 40 years of age and older, again demonstrating how the effect of NF1-associated mortality was mainly restricted to younger individuals, with the difference in survival between individuals with and without NF1 being mostly due to premature mortality from NF1 or NF1-associated causes. However, the finding that there was still a gap of 10 years when considering persons who died at 40 years of age or older suggests that NF1-associated disorders increase the risk of premature mortality also later in life.

As expected, among males, mortality was higher, though not significantly, and the mean age at death was significantly lower. However, the gender difference in age at death was smaller among persons with NF1 (4.4 years) compared to the general population (6.7 years), suggesting that women are affected by more severe NF1-related complications or that NF1 is more likely to be reported on the death certificates of young women. Some studies have shown that women with NF1 have a higher risk of malignancies than males [17,18], though others have not [2,19,20]. However, during pregnancy and puberty, the progression to malignancy and the number and size of neurofibromas may increase in women with NF1 [21], and the greater attention placed on the health of women during pregnancy or childbearing could be reflected in a greater tendency for NF1 to be reported on the death certificates of young women.

The comparison of the two calendar periods may suggest that the survival of persons with NF1 has slightly increased, though longer time series need to be evaluated.

For the leading causes of NF1-associated deaths, we found an excess for malignant neoplasms of connective and other soft tissue and brain, which is not surprising. In fact, NF1 is unlikely to cause death, which is usually caused by NF1-related complications, mainly tumors of the peripheral or central nerve tissue [4-6,9,11,22-28]. In particular, optic gliomas and cutaneous, subcutaneous and plexiform neurofibromas, which are diagnostic of NF1, can become malignant or they can remain benign yet cause pressure on the spinal cord or stenosis and occlusion of cerebral arteries. We also found a significant excess, though smaller or for very few cases, for neoplasm of unspecified parts of the nervous system, neoplasm of ill-defined/unspecified sites, and neoplasm of unspecified nature; when we reviewed these death certificates, we found that all of them mentioned involvement of the brain or nervous system; it is thus likely that the PMRs for malignant neoplasm of brain and connective and soft tissue were underestimated. Moreover, of the 150 deaths with neurofibromatosis recorded as the underlying cause, 17 mentioned lethal complications, and although all of these complications consisted of neoplasm of brain and connective soft tissue, they were not listed as the underlying cause. The decision as to what should be listed as the underlying cause could reflect differences in how individual physicians interpret the hierarchy of causes of death.

The estimated PMRs stratified by age confirmed the weight of premature mortality in NF1-associated deaths due to cancer.

With regard to non-neoplastic diseases, the 2-fold excess for obstructive chronic bronchitis among persons 40 years of age and older is consistent with the well-known respiratory manifestations of NF1. In NF1, the thorax and lungs can be affected in several ways, given the dense distribution of peripheral nerves throughout the thorax: cutaneous and subcutaneous neurofibromas on the chest wall and chest wall deformities; kyphoscoliosis; ribbon deformity of the ribs; thoracic neoplasms; parenchymal neurogenetic tumors [29-31]; and other respiratory manifestations that severely impair pulmonary function, such as upper airway obstruction by neurofibromas, central hypoventilation, diaphragm paralysis, diffuse and interstitial lung disease, and primary pulmonary hypertension [32-35].

The findings of the present study are consistent with those of the only other nationally representative population-based study on NF1 mortality using death certificates, which was conducted by Rasmussen et al. [12], as well as those of smaller survival studies based on the clinical follow-up of persons with NF1, performed in other industrialized countries [36]. In particular, the study by Rasmussen et al. [12], whose methodology was very similar to ours, found a mean annual frequency of NF1-associated deaths of 1/8 700 deaths for the period 1983-1997, which is slightly higher than our estimate of 1/10 685 deaths for a later period, 1995-2006. However, this difference could reflect temporal changes in the mortality of the general population rather than true differences in the survival of the NF1-population. In fact, Rasmussen found a mean age at death of White persons with NF1 of 55.4 years, which is very similar to our estimate of 55.5 years. Furthermore, in both studies, the difference in age at death between men and women was smaller among persons with NF1 compared to the general population. Moreover, the two studies show similar patterns of excesses/deficits for all conditions likely to be an underlying cause (i.e., malignant neoplasms of connective and soft tissue and brain). By contrast, the results differed with regard to other conditions (i.e., curvature of spine, epilepsy and mental retardation), which could be because Rasmussen analyzed all diagnoses reported on the death certificate, whereas we analyzed only the underlying cause.

Some potential limitations of this study need to be considered. Regarding the exclusion of NF1-associated deaths, we are quite confident that any bias would be negligible because the diagnoses were written out (as opposed to being coded), which allowed us to select nearly all death certificates with a diagnosis of NF1 and distinguish it from NF2. We searched for the terms "neurofibromatosis", "Von Recklinghausen disease", and specific symptoms, including synonyms, acronyms and short forms. Thus the only death certificates potentially excluded were those with a diagnosis entered so incorrectly that it eluded the dictionary of medical terms used by ISTAT. Regarding the erroneous inclusion of NF2 cases, as mentioned, NF1 and NF2 can be distinguished because the diagnoses are written out; they can also be distinguished when a diagnosis of neurofibromatosis with no other specification is reported yet the diagnoses of some complications/symptoms specific to NF1 or NF2 are reported. Although it is possible that the 215 cases with a diagnosis of neurofibromatosis without either specification or specific complications/symptoms included some cases of NF2, the mean age at death of these cases did not significantly differ from that of the other 418 cases with NF1, whereas the 23 cases excluded because of an evident or probable diagnosis of NF2 were significantly younger than the selected cases, which is consistent with the greater severity for NF2. Multiple-Causes-of-Death records were missing for the provinces of Trento and Bolzano, yet given that only 3 persons with neurofibromatosis were identified based on the National Mortality Database, this probably did not affect our results. Finally, the quality of data must be considered. Although the automated selection of the underlying cause of death has reduced coding and processing errors [37], the completeness and accuracy of the death certificate and the decedent's medical diagnosis remain as potential sources of error [38,39]. Because physicians report only those conditions and co-morbidities that they believe (or know) to have been instrumental in causing death, it is likely that studies based on death certificates detect only severe NF1-cases, resulting in a potential underestimate of the life expectancy of persons with NF1. It is also likely that only associations with conditions well-known to be associated with NF1 were revealed, resulting in a potentially biased pattern of excesses/deficits for other clinical conditions. Furthermore, the PMR is probably underestimated for conditions that are frequent among NF1 patients and known to be associated with the disease, such as breast cancer, nervous system diseases, and hypertensive disease. This is because these diseases are unlikely to be considered as the underlying cause or to be directly involved in causing death when more aggressive complications are present, and in our study, to study the association between NF1 and other conditions, we were able to consider only the underlying cause (the only available coded cause reported on death certificates).

Finally, death certificates do not allow all persons with NF1 to be identified; thus the association with other conditions should not be interpreted as the risk of developing that condition among living persons with NF1.

## Conclusion

Despite the limits discussed above, this study has important strengths. To the best of our knowledge, it is the first nationally representative population-based study on NF1-associated mortality in Italy. The combined use of the two national data sources allows for comparison with data from the general population and reveals the usefulness of already available data when a surveillance system is not fully operational. This study also stresses the importance of the Multiple-Causes-of-Death Database in providing a more complete picture of mortality for conditions that are frequently not recorded as the underlying cause of death, or to study complex chronic diseases or diseases that have no specific ICD code, such as NF1.

## Abbreviations

CI: Confident Interval; ICD-9: International Classification of Diseases, 9^th ^Revision; ICD-10: International Classification of Diseases, 10^th ^Revision; ISTAT: Italian National Institute of Statistics; NF1: Neurofibromatosis type 1; NF2: Neurofibromatosis type 2; PMR: Proportionate Mortality Ratio; RR: Rate Ratio.

## Competing interests

The authors declare that they have no competing interests.

## Authors' contributions

MM made substantial contributions to conception and design, as well as statistical analysis and interpretation of data, and drafted the manuscript. YK contributed to conception and design, clinical evaluation of death certificates, interpretation of data, and helped to draft the manuscript. MV, SC participated in conception and design and critically revising the manuscript. MK has been involved in drafting the manuscript. MP, LF contributed to acquisition of data and critically revising the manuscript. DT conceived the study and critically revising the manuscript for important intellectual content. All authors read and approved the final manuscript.

## Funding

No funding was provided for this study.
